# Prior Individual Training and Self-Organized Queuing during Group Emergency Escape of Mice from Water Pool

**DOI:** 10.1371/journal.pone.0118508

**Published:** 2015-02-18

**Authors:** Caesar Saloma, Gay Jane Perez, Catherine Ann Gavile, Jacqueline Judith Ick-Joson, Cynthia Palmes-Saloma

**Affiliations:** 1 National Institute of Physics, College of Science, University of the Philippines, Diliman, Quezon City, Philippines; 2 National Institute of Molecular Biology and Biotechnology, College of Science, University of the Philippines, Diliman, Quezon City, Philippines; Universidad Carlos III de Madrid, SPAIN

## Abstract

We study the impact of prior individual training during group emergency evacuation using mice that escape from an enclosed water pool to a dry platform via any of two possible exits. Experimenting with mice avoids serious ethical and legal issues that arise when dealing with unwitting human participants while minimizing concerns regarding the reliability of results obtained from simulated experiments using ‘actors’. First, mice were trained separately and their individual escape times measured over several trials. Mice learned quickly to swim towards an exit–they achieved their fastest escape times within the first four trials. The trained mice were then placed together in the pool and allowed to escape. No two mice were permitted in the pool beforehand and only one could pass through an exit opening at any given time. At first trial, groups of trained mice escaped seven and five times faster than their corresponding control groups of untrained mice at pool occupancy rate *ρ* of 11.9% and 4%, respectively. Faster evacuation happened because trained mice: (a) had better recognition of the available pool space and took shorter escape routes to an exit, (b) were less likely to form arches that blocked an exit opening, and (c) utilized the two exits efficiently without preference. Trained groups achieved continuous egress without an apparent leader-coordinator (self-organized queuing)—a collective behavior not experienced during individual training. Queuing was unobserved in untrained groups where mice were prone to wall seeking, aimless swimming and/or blind copying that produced circuitous escape routes, biased exit use and clogging. The experiments also reveal that faster and less costly group training at *ρ* = 4%, yielded an average individual escape time that is comparable with individualized training. However, group training in a more crowded pool (*ρ* = 11.9%) produced a longer average individual escape time.

## Introduction

Experiments in crowd emergency evacuation are difficult to perform under genuine settings particularly with human participants [1–4]. Deliberately exposing unwitting pedestrians to extreme danger is unlawful and ethically questionable. However, incidents with unacceptably high casualties continue to happen [5–6] and the urgency remains to improve further our current understanding of emergency evacuation dynamics. The task is hard since crowds are complex social systems that exhibit a range of possible collective behavior not easily deduced from the individual performance of members [7–12].

Crowd emergency evacuation could happen in a variety of physical systems including mass transportation, enclosed public places, threatened ecosystems, calamity-stricken communities [5–6] and financial institutions [13]. Concerned individuals need to decide soundly and act quickly in order to survive—an extremely difficult task to accomplish in the presence of others whose first priority is their own personal safety. Researchers from different disciplines have been searching for better evacuation strategies that achieve optimal balance between design and functionality as well as structural integrity and fiscal viability [7].

Cooperation, orderly conduct and good signage visibility contribute greatly in the efficient utilization of available exits under normal circumstances. However, self-regulation is hard to practice in a large group of interacting strangers that is subjected to a set of rapidly deteriorating ambient conditions. Large-scale emergency response exercises are generally useful but they are also time-consuming and costly to conduct. Hence, there is an economic incentive to learn more about the tangible benefits of drills and training exercises. For example, a better understanding of the learning rates of trainees is valuable in minimizing over-training that yields diminishing returns with more drill time.

We might learn more about the dynamics of group emergency evacuation by studying the collective behavior of interacting animals under duress [14–15]. Such studies could generate additional real-world data that might prove useful in testing design hypotheses and evacuation models [2, 16–21]. Here we investigate the influence of prior individual during group emergency evacuation of strangers using mice that escape from a water pool. Experimenting with mice avoids the serious ethical and legal issues that arise when dealing with unwitting human participants. It also minimizes possible concerns regarding the reliability of results that are obtained from simulated experiments using ‘actors’.

## Methodology

**Fig 1:**
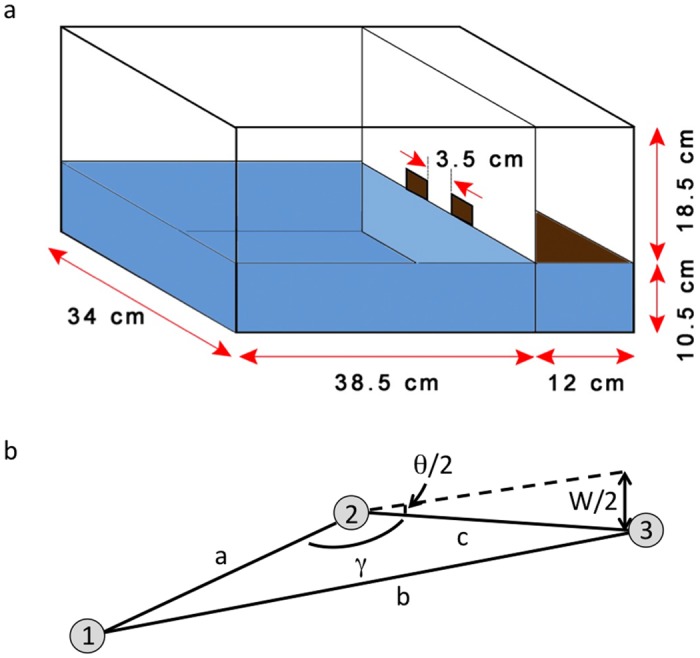
Experimental set-up. (a) Pool dimensions: *L* = 38 cm, *W* = 34 cm, *H* = 10.5 cm = pool depth. Other parameters: Water temperature = 25°C, Exit separation: 3.5 cm, exit width *w* = 3.5 cm, Container dimensions (*L* = 50 cm, *W* = 36 cm, *H* = 28.5 cm) divided in a wet—dry (3:1) configuration. (b) Schematic for determination of escape route trajectory produced by mouse from its release point. Three possible positions (points 1, 2 and 3) of mouse within time duration *Δt* are established from three successive video frames equally sampled at *Δt*/2 where angle *θ*/2 = tan^-1^(*w/2c*).

We study the dynamics of group emergency evacuation using mice that escape from an enclosed water pool to a dry platform via any of two adjacent exits [Fig. 1(a)]. Mice exhibit strong motivation to escape from water (S1 Video). They always attempt to reach dry land in every trial run without additional motivation [14, 22–23].

### Ethics Statement

Naïve male ICR mice were brought from the Research Institute of Tropical Medicine in Muntinlupa City, Philippines and maintained at the Laboratory of Molecular and Cell Biology, National Institute of Molecular Biology and Biotechnology, University of the Philippines (UP) Diliman. They were treated consistent with the guidelines for the suitable treatment of laboratory animals as specified in Administrative Order No. 40 (Department of Agriculture, Government of the Philippines) and the Philippine Animal Welfare Act of 1998. We confirm that the UP Diliman Institutional Animal Care and Use Committee specifically approved this study.

### Training Method

First, we put individual male ICR mice in a two-exit water pool and measure their individual escape times *T(N)* over successive trials *N* to obtain their average individual escape time per trial, *‹T(N)›*. The rate in which *T* changes with *N* indicates the ability of a mouse to develop the skill to escape from the pool and survive.

Next, we place the trained mice together in the pool and then measure the corresponding group evacuation time *T*_*G*_ *(N)*. Different group sizes were tested to determine the merit of prior individual training as a function of pool occupancy rate *ρ*. The mice were initially unfamiliar with each other since no two of them were allowed in the pool before the first group trial (*N* = 1). For comparison, we also determined the evacuation performance of similarly sized control groups composed of mice that received no prior individual training.

To minimize infighting when in groups, we only deployed naïve male ICR mice (age range: 8–16 weeks) that were reared at 25°C with 12-h light/12-h dark periods. Their mean age when first used (*N* = 1) was nine weeks with sizes (cross-section diameter) ranging from 2.5 to 3.0 cm. ICR mice achieve sexual maturity at an age of 7–8 weeks.

**Pool Experiments**. Mice were released individually into an enclosed water pool [length *L* = 38 cm, width *W* = 34 cm, height *H* = 10.5 cm = pool depth; water temperature = 25°C] with two adjacent exits (side separation: 3.5 cm, along the *W*-side) to a dry platform in one end of a rectangular container (*L* = 50 cm, *W* = 36 cm, *H* = 28.5 cm) that is divided in a wet (pool)-dry (3:1) configuration [Fig. 1(a)]. Immediately upon release, a mouse instinctively searches for a way to get off and avoid drowning (S1 Video). The exit opening measures 12.25 cm^2^, which is equivalent to the average cross-section of the mice. Only one mouse could pass through an opening at any given time.

A total of 140 mice were trained individually with their corresponding individual evacuation times *T(N)* measured at 10-minute intervals for *N* ≤ 7. No mouse was left to bathe in the pool for more than 5 minutes—swimming consumes energy rapidly and is the main cause of mouse fatigue. The 10-minute intervals between trials allowed the mice to regain sufficient strength to escape with same vigor when returned to the pool.

Forty-eight hours after individual training, the mice were released as a group and the corresponding group evacuation times *T*_*G*_ *(N)* were taken where *T*_*G*_ is the total time needed for at least 80% of the mice to get off the pool for a given trial. The experiments were done in two group sizes (10, 30 members) with three groups representing each size. The 10-mouse and 30-mouse groups were estimated to represent a pool occupancy rate *ρ* of 4% and 11.9%, respectively [14]. For comparison, *T*_*G*_ *(N)* measurements were also made under the same experimental conditions, on control groups of mice with no prior individual training.

The experiments involved a total of 250 trained and untrained mice and the trials were conducted from May 2005 to June 2006 between 1000 and 1500 hours of the day (local time). The video footages were recorded with a digital camera (Sony Digital Handycam DCR-TRV820 NTSC, digital sampling rate = 25 frames/sec) for post-detection processing.

**Tracking of Mouse Movements in the Pool: Individual mouse**. We monitored the movement of a mouse in the pool by manually tracking the position of its snout in the recorded video footages. We chose the snout since its position is easily identifiable and relatively robust against changes in the mouse body orientation.

The mouse was initially released at a random location that is relatively far from the exits. The shortest escape route from the release point to an exit is a single straight line. The route becomes longer when the moving mouse makes a turn along the way and deviates from following a single straight-line path to an exit. The corresponding route trajectory consists of (*P* + 1) connected line segments of varying lengths where *P* is the number of turns made by the mouse.

Fig. 1(b) illustrates the calculation of the length of the path taken by a moving mouse within time duration *∆t* covering three successive video frames that are equally sampled at intervals of *∆t/*2. We identify the respective pool positions (points 1, 2 and 3) of the mouse from the three frames starting with the release point for the first frame (*t* = 0). These positions are then used to calculate for the values of angles *γ* and *θ*/2 where *θ*/2 is the upper bound of the angular deviation that is allowed for the path from point 1 to 3 to be considered as a single line segment, and *γ* is the angle subtended by the lines joining points 1 and 2 (length *a*), and points 2 and 3 (length *c*), respectively.

Angle *θ*/2 is calculated using: tan(*θ*/2) = *w/2c*, where *w* is the exit width. If 180°—*γ* ≤ *θ*/2, then the path taken is represented by only one line segment of length *b* the distance between points 1 and 3. On the other hand, if 180°—*γ* > θ/2, then the path consists of two line segments of lengths *a* and *c*, respectively. This case happens when the mouse turns at point 2 in order to reach point 3. We repeat the procedure for the succeeding mouse positions (point 3) until the escape route of the mouse to an exit is fully tracked.

**Groups of Mice**. Manual tracking is difficult to perform in groups of same looking mice that are swimming simultaneously. To track a group member we developed an automatic trajectory-search program based on the particle-tracking algorithm [24] that was previously used to follow the flow of identical particles in colloidal suspensions [25].

We identify the locations of each mouse in the group by establishing the coordinates of its apparent (body) geometric center that is chosen instead of the snout since the latter is difficult to locate continuously—mice could swim over each other and overlapped their bodies. The different positions of the mice are tracked in time by locating their geometric center coordinates.

The tracking algorithm locks-in to the escape route trajectory comparing the mouse center coordinates between successive frames. It finds pairs of coordinates with minimum differences and identifies such pairs as consecutive positions of a single mouse. The algorithm is accurate if the average displacement of the mouse is less than the inter-mouse distance. When a mouse suddenly disappears from the field of view of the camera, for example by submerging into the pool, a memory of its last observed location is kept. If it reappears on the same spot at a later time then it is recognized as the same mouse that previously disappeared. However, it could be identified as a different mouse if it surfaces at a location that is far from where it first vanished. Two trajectories are difficult to discriminate from each other through time when the two mice that produced them moved close to each over extended time periods which is more likely at high room occupancy rates.

## Experimental Results and Data Analysis

We found that the average individual escape time *‹T(N)›* decreases quickly with *N* and that physical fatigue limited the capacity of mice to endure longer training periods beyond *N* = 7. Trained mice responded more effectively against the persistent threat of drowning. At *N* = 1, they escaped *7x* and *5x* faster than their untrained counterparts at pool occupancy rates of *ρ* = 11.9% and 4%, respectively.

We also analyzed the underlying egress dynamics that enabled trained groups to escape more quickly by determining the routes taken by their individual group members towards an exit location. We measure the group evacuation times *T*_*G*_ *(N)* and check for evidence that indicate optimal use of the two available exits.

### Escape Route Trajectories in Water Pool

We calculated the escape trajectory of a mouse whether left alone or with others using a set of video frames that are equally sampled at intervals of *∆t/*2 = 0.125 second (8 frames/sec). The said *∆t/*2 value provided us with an acceptable balance between tracking accuracy and trajectory computation time. A trajectory may be calculated at a higher spatial resolution with more video frames sampled at a smaller *∆t/*2 value at the expense of a longer computation time. With our video camera, the smallest value available is *∆t/*2 = 0.04 sec.

**Fig 2:**
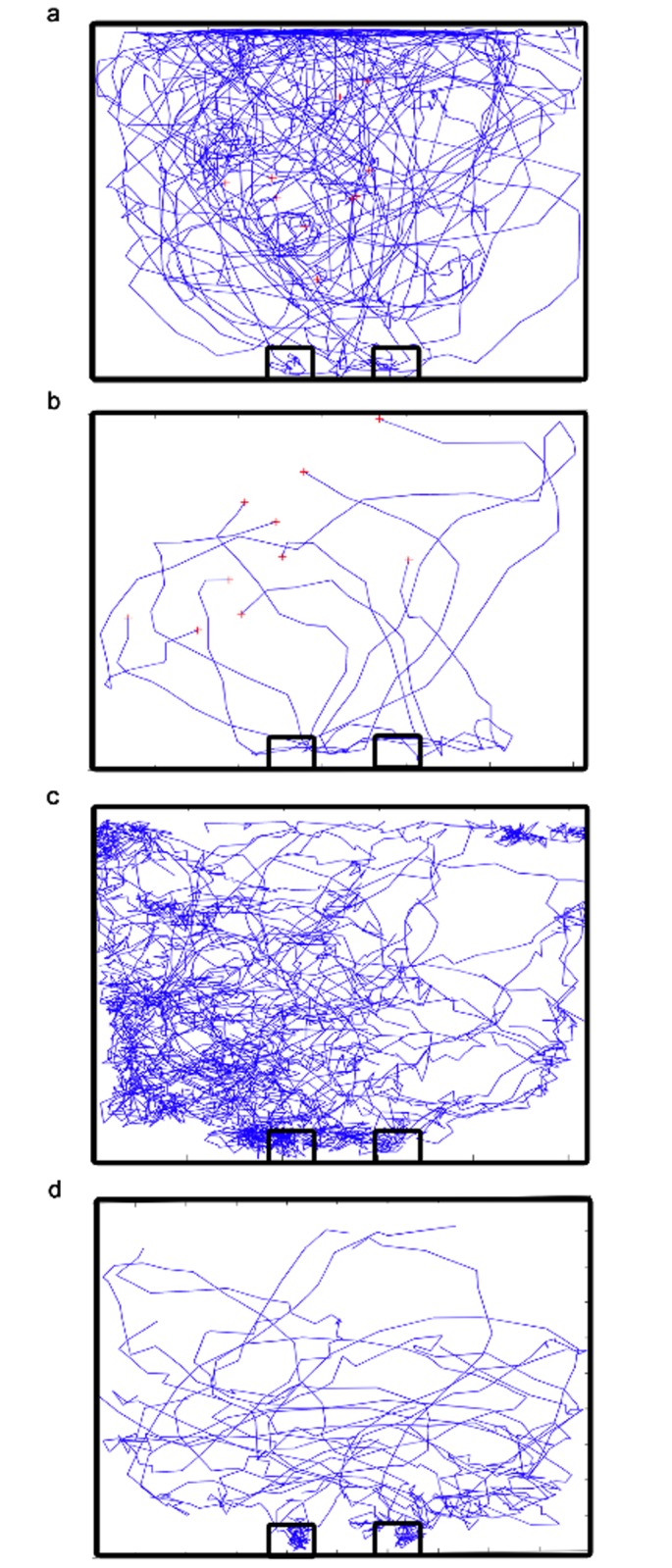
Escape route trajectories of mice from release points to an exit. Superposed trajectories produced by 10 mice undergoing individual training at (a) *N* = 1 and (b) *N* = 7. Trajectories produced by a group of: (c) 10 untrained and (d) 10 individually trained mice at *N* = 1. Crosshairs indicate the release points of individual mice at *t* = 0. Boxes specify the exit locations. Mice in a group are released together far from the exit side. Each trajectory is calculated from a set of video frames separated at 0.125-second intervals.

**Individual Mouse**. Presented in Fig. 2(a) is a superposition of the escape trajectories produced by ten representative mice during individual training at *N* = 1. The release points at time *t* = 0 are marked with crosshairs and the two exit locations are indicated by small boxes. Locations with high line densities indicate pool areas that were visited more often by mice. Initially (*N* = 1), a mouse would swim aimlessly and exhibit wall-seeking behavior causing it to stay longer in the pool. The average time that the 10 untrained mice spent in the pool is *‹T(1)›* = 46 ± 26 sec (*N* = 1).

Fig. 2(b) shows the superposed trajectories produced by the same ten mice at *N* = 7. The trajectories are now shorter and less densely distributed than those produced at *N* = 1. By *N* = 7, the individual mice already acquired a good ‘sense’ of the general exit locations immediately after release. Their average individual escape time improved considerably to *‹T(7)›* = 3.29 ± 0.84 sec. In Figs. 2(a) and 2(b), the ten trajectories produced by the mice are found over the entire pool area towards the general exit direction.

**Groups of mice**. Fig. 2(c) presents the escape trajectories produced by a group of 10 untrained mice [*ρ* = 4%, *N* = 1, group escape time *T*_*G*_ *(1)* = 160 sec] that were released simultaneously at a pool location that is far from the exit side. The mice moved to common locations as indicated by the denser line distributions in certain pool areas. Note that the left side of the pool is more utilized than the right. They were unable to utilize the two exits efficiently—the left exit is targeted more frequently than the other. Fig. 2(c) shows that the trajectories are unevenly distributed unlike those in Fig. 2(a) that are due to mice swimming alone in the pool.

Fig. 2(d) presents the trajectories produced by a group of 10 individually trained mice [*ρ* = 4%, *T*_*G*_ *(1)* = 27 sec]. The trajectories are shorter and less dense than those shown in Fig. 2(c). The trained mice escaped faster as a group spending only about one-fifth the amount of time in water than their untrained counterparts. They utilized the available pool space and exits more efficiently as indicated by the even distribution of the escape trajectories toward the exits.

We also tested larger groups of 30 individually trained mice (*ρ* = 11.9%) and compare their performance with corresponding control groups of 30 untrained mice. The calculated trajectories exhibit the same characteristics as those shown in Figs. 2(c) and 2(d) for the untrained and trained groups, respectively. However, the trajectory distributions are a lot denser and are no longer presented here. The benefit of prior individual training becomes clearer in a more crowded pool where groups of thirty trained mice (*ρ* = 11.9%) escaped *7x* faster than their corresponding untrained counterparts.

### Evacuation Skill

**Fig 3:**
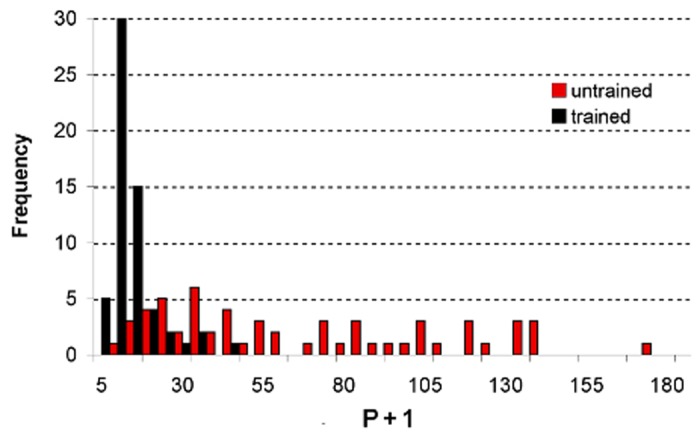
Number of turns *P* made by individual mice on their way to an exit. Escape time is fastest when a mouse goes straight to an exit without turning (*P* = 0). Escape trajectory from a release point to an exit consists of (*P* + 1) connected line segments. Sampling size: 60 trained and 60 untrained mice.

**Number of turns made by individual mice to reach an exit**. Fig. 3 presents a histogram of the number of turns *P* that individual mice took to reach an exit from their initial release points. Escape is fastest when a mouse follows a single straight route towards to an exit without turning (*P* = 0). None in the cohort of 60 individually trained mice made more than 35 turns—83.3% of them only took 15 turns or less. On the other hand, the histogram produced by a cohort of 60 untrained mice is broader—75% of them took at least 35 turns each (range: 35–175 turns) before escaping. Untrained mice followed circuitous trajectories composed of a large number of (*P* + 1) connected short-line segments. In all cases, no mouse was able to leave the pool without making at least one turn (*P* = 1).

**Fig 4:**
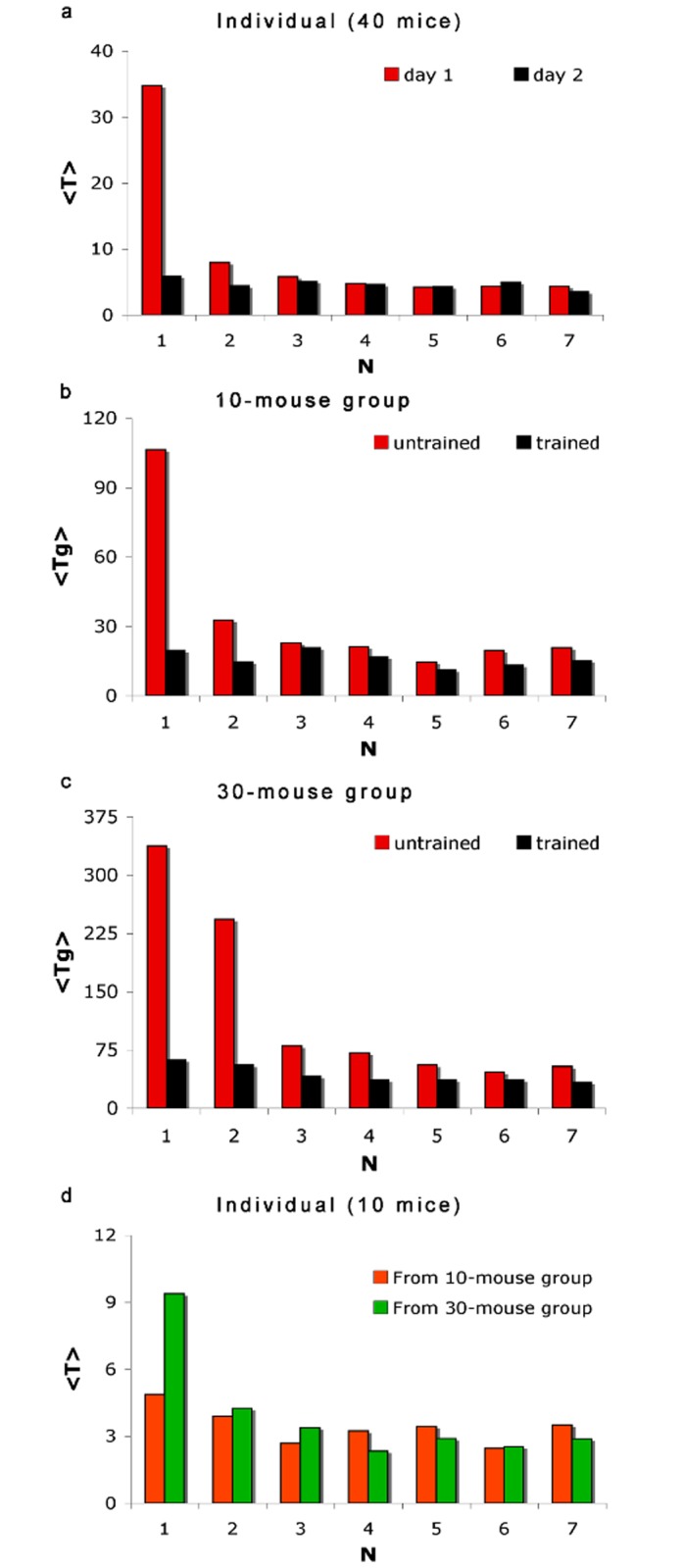
Evacuation Performance. Average escape time (in sec) against trial number *N* obtained from: (a) Individual training of 40 mice (red), and 24 hours after training (black), (b) three 10-mouse groups, and (c) three 30-mouse groups. Shown in (D) is the average individual escape time *‹T(N)›* of ten mice with no prior individual training, taken 6 weeks after participating in group trials.

Fig. 4(a) plots the average individual escape time *‹T(N)›* achieved by 40 individually trained mice as a function of trial number *N* ≤ 7. Mice achieved their fastest individual escape times within the first four trials and the acquired skill is long-term—24 hours after training, the ‘rested’ mice still performed with the same escape times.

Fig. 4(b) plots the average group escape time *‹T*_*G*_ *(N)›* produced by three groups of 10 individually trained and untrained mice (*ρ* = 4%), for *N* ≤ 7. At *N* = 1, the trained groups clocked faster *T*_*G*_ values than their untrained counterparts that were also able to gradually improve their group evacuation performance within the first 4 trials. Group performance for both groups showed signs of deterioration starting at *N* = 5 due to physical fatigue.

Similar performance characteristics were also observed in larger 30-mouse groups [Fig. 4(c)]. In a more crowded pool (*ρ* = 11.9%), mice were more susceptible to physical exhaustion since they stayed longer in water and exerted more effort to approach an exit in the presence of more competitors.

For groups of 10 trained mice, *‹T*_*G*_ *›* ~ 10*‹T(7)›*, implying that even in the presence of others competing for an exit opening, individually trained mice were still able to perform near to their best individual ratings by efficiently utilizing the entire pool space and the two exits for escape.

We also investigated the effect of group escape experience by measuring the individual escape times *T(1)* of participating mice with no prior individual training. Fig. 4(d) reveals that the said mice were able to acquire useful evacuation skills particularly if trained in smaller 10-mouse groups (*ρ* = 4%). They clocked an average individual escape time *‹T(1)›* ~ 4.7 sec, that is comparable with that achieved through time-consuming individualized training. However, training in a more crowded pool (*ρ* = 11.9%) yielded a longer average escape time *‹T(1)›* ~ 9.2 sec.

### Self-organized queuing

We determine the number of mice that escaped through either exit as a function of time by counting the corresponding number that remained in the pool at sampling intervals of 2 seconds, which is the average time needed by a mouse to completely cross an exit opening to the dry platform.

**Fig 5:**
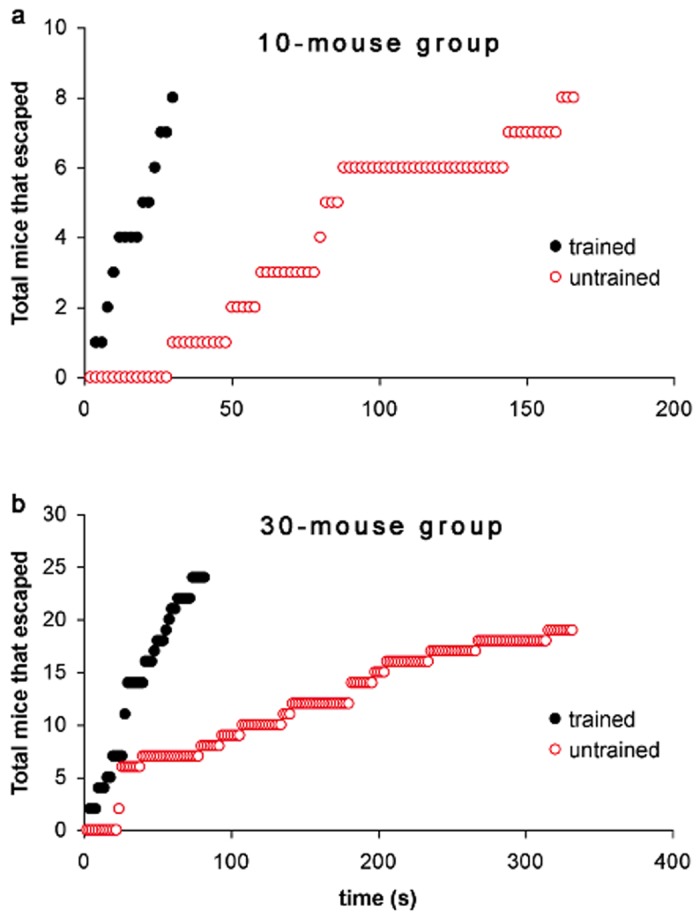
Self-organized queuing. Total number of mice that escaped as function of time (*N* = 1) in: (a) 10-mouse group, and (b) 30-mouse group. The number of trained mice in the pool decreases linearly with time.

Fig. 5(a) shows that a group of ten individually trained mice (*N* = 1, solid black circles) were able to egress continuously causing the number of mice remaining in the pool to decrease linearly with time. Eighty percent of the trained mice were able to escape within the first 30 seconds of release. We term this phenomenon of continuous egress as self-organized queuing.

On the other hand, the corresponding groups of ten untrained mice (*N* = 1, red circles) were unable to leave as quickly as the trained groups and their members stayed longer in the pool. Untrained mice escaped in punctuated bursts of different sizes characterized by periods of time (one lasting for 56 seconds) when not one was able to escape. No untrained mouse escaped in the first 35 seconds and only two in first 50 seconds after their release.

Similar egress characteristics were observed in larger 30-mouse groups. Only about 65% of the untrained mice were able to escape within the first 350 seconds [Fig. 5(b)]. Trained mice on the other hand, streamed out of the exits continuously in time—80% of them were already on dry platform within the first 75 seconds of release. Queuing was observable regardless of the video-frame interval (1, 2 or 5 seconds) used in determining the number of mice remaining in the pool. It was not observed in untrained groups.

## Discussion and Conclusions

The benefit of prior individual training during group emergency evacuation became more tangible as the pool got more occupied. At *N* = 1, groups of individually trained mice escaped *7x* and *5x* faster than their untrained counterparts at *ρ* = 11.9% (30 mouse groups) and 4% (10-mouse groups), respectively.

Faster group evacuation happened because trained mice: (a) had better recognition of the available pool space and took shorter routes to an exit, (b) were less likely to form arches that blocked an exit opening, and (c) utilize the two exits efficiently without preference. Continuous egress (queuing) is a collective behavior that is not possible during individual training. It was not observed in untrained groups at *N* = 1 where mice were prone to wall seeking, aimless swimming and blind copying (imitation)—traits that have produced circuitous escape routes as well as biased exit use and clogging.

The occurrence of blind copying is suggested by the uneven (biased) utilization of the available pool space and exits by untrained members especially in the larger 30-mouse groups [Fig. 2(c)]. Mice did not exhibit such kind of bias during individual training at *N* = 1 [Figs. 2(a) and 2(b)]. Blind copying promotes herding behavior in animal groups often with dire consequences to participants [8, 26]. With more mice competing for one and the same exit opening, clogging by arching became more likely especially in a narrow opening that allowed just one mouse to pass through at a given time. With preferential use, the availability of another nearby exit was disregarded as an alternate avenue for escape. It illustrates the critical role that collective animal behavior could play in the design (e.g. number and locations of exits) of an effective architecture for emergency evacuation.

Our results show that prior individual training favors the emergence of self-organized queuing during group emergency escape. Self-organized queuing happened since the trained mice were able to coordinate with each other to a certain degree possible even in the absence of a leader-coordinator. Coordination develops when group members are able to commit information-driven actions instead of blind copying or non-copying (e.g. aimless swimming) [8, 26–27].

We found that long-term individual evacuation skills could be acquired through group (batch) training, which is faster and less tedious to hold than individualized training. Members from 10-mouse groups (*ρ* = 4%) when tested six weeks after group trials, yielded an average individual escape time *‹T(1)›* that is comparable with that achieved by individualized training [Fig. 4(d)]. However, training in a more occupied pool (*ρ* = 11.9%) with the 30-mouse groups yielded a larger *‹T(1)›* value.

Our experiments with mice revealed a number of interesting dynamical features of group emergency evacuation involving strangers. Our findings on the influence of prior individual and small-group training may lend useful insights on the positive attributes of drills and exercises in other similar cases involving other species including humans. Individual mice learned within the first four trials and the introduction of more training time was superfluous. The acquired evacuation skills were long term and lasted for at least six weeks. Together, individually trained mice were less prone to blind copying and displayed better recognition of available pool space and exits. They did not crowd around a particular exit thereby reducing the likelihood of clogging. The said factors paved the way for quicker group evacuation through self-organized queuing.

## Supporting Information

S1 Video Emergency escape of mice from water pool. (MOV)

## References

[1] Bryan J. A Selected Historical Review of Human Behavior in Fire. Fire Prot Eng. 2002;16: 4–10.

[2] Helbing D, Buzna L, Johansson A, Werner T. Self-Organized Pedestrian Crowd Dynamics: Experiments, Simulations, and Design Solutions. Transportation Sci. 2005;39: 1–24.

[3] Helbing D, A Johansson A, Al-Abideen H. Dynamics of crowd disasters: An empirical study. Phys Rev E. 2007;75: 046109 17500963 10.1103/PhysRevE.75.046109

[4] Levitt S, List J. Homo economicus Evolves. Science. 2008;319: 909–910. doi: 10.1126/science.1153640 18276876 10.1126/science.1153640

[5] Still GK. Crowd Disasters. Available: www.gkstill.com/ExpertWitness/CrowdDisasters.html

[6] EMDAT: Emergency Events Database. Available: www.emdat.be/.

[7] Waldau N, Gatterman P, Knoflacher H, Schreckenberg M, editors. Pedestrian and Evacuation Dynamics 2005. Berlin: Springer; 2007.

[8] Perez GJ, Saloma C. Allelomimesis as escape strategy of pedestrians in two-exit confinements. Physica A. 2009;388: 2469–2475.

[9] Nolfi S. Behavior as a complex adaptive system: On the role of self-organization in the development of individual and collective behavior. Complexus. 2004/2005:2; 195–203.

[10] Castellano C, Fortunato S, Loreto V. Statistical physics of social dynamics. Rev. Mod. Phys. 2009;81: 591–646.

[11] Helbing D, I. Farkas I, Vicsek T. Dynamical features of escape panic. Nature. 2000;407: 487–490. 11028994 10.1038/35035023

[12] Ball P. The Physical Modelling of Human Social Systems. Complexus. 2003;1: 190–206.

[13] Sobel R. Panic on Wall Street: A Classic History of America’s Financial Disasters-With a New Exploration of the Crash of 1987. Boston: E P Dutton; 1988.

[14] Saloma C, Perez GJ, Tapang G, Lim M, Palmes-Saloma C. Self-organized queuing and scale-free behavior in real escape panic. Proc Natl Acad Sci USA. 2003;100: 11947–11952. 14519853 10.1073/pnas.2031912100 PMC218693

[15] Sumpter D. The principles of collective animal behaviour. Phil. Trans. Royal Soc. Lond. B. 2006;361: 5–22. 16553306 10.1098/rstb.2005.1733 PMC1626537

[16] Helbing D. Traffic and related self-driven many-particle systems. Rev. Mod. Phys. 2001;73: 1067–1141.

[17] Perez GJ, Tapang G, Lim M, Saloma C. Streaming, disruptive interference and power-law behavior in the exit dynamics of confined pedestrians. Physica A. 2002;312: 609–618.

[18] Schadschneider A, Klingsch W, Klupfel H, Kretz T, Rogsch C, Seyfried A. Evacuation dynamics: Empirical results, modeling and application In: Meyers R, editor. Encyclopedia of Complexity and System Science. Berlin: Springer; 2009 pp 3142–3176.

[19] Zhao D, Yang L, Li J, Zou L. Relationship between Performance-based Design of Building Exits and State Transition of Pedestrian Flow during Occupant Evacuation. J Fire Prot Eng. 2006;16: 269–281

[20] Arita C, Schadscheneider A. Dynamical analysis of the exclusive queuing process. Phys Rev E. 2011;83: 051128 21728511 10.1103/PhysRevE.83.051128

[21] Bode N, Codling E. Human exit route choice in virtual crowd evacuations. Animal Behaviour. 2013;86: 347–358

[22] Wenk G. In: Crawley J, Gerfen C, McKay R, Rogawski M, Sibley D, Johansson H, editors. Current Protocols in Neuroscience. New York: Wiley; 1998 pp 8.5A.1–8.5A.12

[23] Morris R. Developments of a water-maze procedure for studying spatial learning in the rat. Neurosci. Method. 1984;11: 47–60 6471907 10.1016/0165-0270(84)90007-4

[24] Weeks E, Crocker J. Available: http://www/physics.emory.edu/~weeks/idl/

[25] Crocker J, Grier D. Methods of Digital Video Microscopy for Colloidal Studies. J Colloid Interface Sci. 1996;179: 298–310.

[26] Juanico D, Monterola C, Saloma C. Cluster formation by allelomimesis in real-world complex adaptive systems. Phys. Rev. E. 2005;71: 041905 15903699 10.1103/PhysRevE.71.041905

[27] Parrish J, Edelstein-Keshet L. Complexity, Pattern, and Evolutionary Trade-Offs in Animal Aggregation. Science. 1999;284: 99–101. 10102827 10.1126/science.284.5411.99

